# Distribution of benzalkonium chloride into the aqueous phases of submicron dispersed systems: emulsions, aqueous lecithin dispersion and nanospheres

**DOI:** 10.1208/s12249-019-1540-7

**Published:** 2019-12-02

**Authors:** Dorota Watrobska–Swietlikowska

**Affiliations:** Department of Pharmaceutical Technology, Medical University of Gdansk, Hallera Av. 107, 80-416 Gdansk, Poland

**Keywords:** Submicron emulsion, Preservatives, Benzalkonium chloride, Aqueous lecithin dispersion, Nanospheres

## Abstract

Partitioning of benzalkonium chloride (BAC) into the aqueous phases of submicron dispersed systems such as submicron emulsions, aqueous lecithin dispersion (WLD), and suspension of nanospheres (NLC) was studied. The aqueous phases of the investigated systems were obtained by ultracentrifugation and subsequently were subjected to ultrafiltration, which procedure allowed distinguishing between the fractions of free benzalkonium chloride (*w*) and those incorporated in the liposomal and micellar region (*wlm*). The fractions present in the oily phase and in the interphase of submicron emulsions were calculated. Despite the various composition of the investigated formulations and the initial concentration of BAC, *w* values were very small at 0.2–8.0%. The *wlm* value in submicron emulsions was increased by increasing the total concentration of preservative from 29.0 to 42.0%. Using polysorbate 80 instead of lecithin resulted in a distribution of BAC to aqueous–liposomal–micellar phase that was twice as high. The very low concentration of antimicrobial active form of benzalkonium chloride was analyzed in the aqueous phase of emulsions stabilized with lecithin as well as in aqueous lecithin dispersion and nanospheres (below 3%). Replacement of lecithin with polysorbate 80 in emulsions with polysorbate significantly increase (up to 8%) the fraction of benzalkonium chloride in the aqueous phase where microbial growth occurs.

## INTRODUCTION

Pharmaceutical and cosmetic products that contain the aqueous phase should be properly preserved against microbial contamination and proliferation during storage in normal conditions and proper use (1).

Submicron dispersions are modern drug delivery systems for poorly soluble drugs. As so far, submicron emulsions are commonly used in parenteral nutrition admixtures, but aqueous lecithin dispersion and nanospheres are still potential alternatives for drug delivery particles. Preservation of submicron dispersed systems is a very important problem and there are few articles about the preservation of submicron emulsions with parabens (2–4). There is no publication about the preservation of submicron dispersed systems using more safe preservative as benzalkonium chloride. Additionally, preservation of submicron dispersed systems is a more challenging task due to a more complex internal structure, the existence of different phases, and the expanded interphase (5). Due to the complex internal structures, preservatives of these systems may not attain an effective concentration in the aqueous phase. Unfortunately, there is only limited research on this problem which is important for the use of modern formulation in clinical practice.

The preservative should protect the aqueous phase where the microbial growth occurs and it is important to choose the proper preservatives and their concentration for each formulation (6,7). Most preservatives are lipophilic so it is difficult to obtain the appropriate concentration in the aqueous phase. Benzalkonium chloride is a preservative which possesses properties of cationic surfactant. Benzalkonium chloride is very soluble in water, ethanol, and acetone (8). Aqueous solutions of benzalkonium chloride foam when shaken have a low surface tension, detergent, and emulsifying properties.

Lecithin as a biocompatible emulsifier is used in submicron dispersed systems intended for parenteral use (9–11). However, lecithin in high concentration is used for inactivation of preservatives in the pharmacopeial sterility test. For this reason, the important aspect of this investigation was to determine the influence of lecithin phospholipids on the distribution of preservatives and to compare them with formulations stabilized with another surfactant—polysorbate 80.

Benzalkonium chloride is a quaternary ammonium compound used in pharmaceutical formulations as an antimicrobial preservative (8). Benzalkonium chloride, also known as alkyldimethylbenzylammonium chloride, is a mixture of alkylbenzyldimethylammonium chlorides of various even-numbered alkyl chain lengths. Benzalkonium chloride is one of the most widely used preservatives in ophthalmic preparations (12). It is used in nasal and otic formulations (13) and as well as in small-volume parenteral products. This preservative is additionally used as preservatives in cosmetics (14).

Nowadays, there is a tendency to avoid preservatives in ophthalmic preparation because all preservatives, not only benzalkonium chloride (BAC), are not safe for the ocular surface. However, as it was mentioned, BAC is the most popular preservative in ophthalmic preparation (nearly 75% of ophthalmic preparation contains BAC) and if we say that the efforts have been put into preservative-free formulations that have replaced formulations that contain preservatives, meaning mainly BAC. It is noteworthy to mention that in parenteral small-volume multidose preparations, the presence of preservatives is essential and BAC characterizes effective bactericidal and fungicidal properties that help to minimize the growth of organisms in multidose containers. That was the reason for choosing BAC as a preservative in this study.

The greatest biocidal activity of benzalkonium chloride is associated with the C12–C14 alkyl derivatives. The mechanism of microbicidal action is thought to be due to the disruption of intermolecular interactions (8). This can cause dissociation of *cellular membrane lipid bilayers*, which compromises cellular permeability controls and induces leakage of cellular contents. Benzalkonium chloride solutions are active against bacteria and some viruses, fungi, and protozoa. Bacterial spores are considered to be resistant. Solutions are *bacteriostatic* or *bactericidal* according to their concentration. *Gram*-*positive* bacteria are generally more susceptible than *Gram*-*negative* (8).

Benzalkonium chloride is usually nonirritating, nonsensitizing, and well tolerated in the dilutions normally employed on the skin and mucous membranes. However, this preservative has been associated with adverse effects when used in some pharmaceutical formulations. Ototoxicity or ocular toxicity of benzalkonium chloride is associated with long-term exposure of this preservative (15). Other data suggest that benzalkonium chloride may produce adverse clinical effects on human nasal tissue, in patients using nasal spray (15). Benzalkonium chloride is also known to cause bronchoconstriction in some asthmatics when used in nebulizer solutions (16). It also has *cytotoxic effects* in human cells and causes adverse effects such as *dermatitis*, due to the frequent use of antiseptics by healthcare workers (17).

Solutions of benzalkonium chloride are stable over a wide pH and temperature range and may be sterilized by autoclaving without a loss of effectiveness (8). Dilute solutions stored in polyvinyl chloride or polyurethane foam containers may lose antimicrobial activity (18). Benzalkonium chloride is incompatible with aluminum, anionic surfactants, nonionic surfactants in high concentration, hydrogen peroxide, iodides, kaolin, nitrates, silver salts, soaps, and also some rubber and plastic mixes (19).

The aim of the study was to investigate the compatibility of benzalkonium chloride with parenteral dispersed systems such as emulsions, aqueous lecithin dispersion, and suspension of nanospheres. The second aim was to evaluate the distribution of benzalkonium chloride between phases of drug-free submicron dispersion in relation to the oily phase and lecithin presence as well as to the benzalkonium chloride concentration. The studies allow estimating if benzalkonium chloride can be an alternative preservative of modern dispersed systems.

## MATERIALS AND METHODS

### Preparation of dispersed systems

Submicron emulsions (E0, E1, E2, E3, EP0, EP) were prepared according to a standard method employing a hot-stage high-pressure homogenization, 8 cycles at 500 bar (20). They consisted of soya bean oil (10% w/w) and (1.2% w/w) egg lecithin Lipoid E-80 (Lipoid, Ludvigshafen, Germany) for emulsion E0 and E1–E3 or polysorbate 80 (Sigma, St. Louis, USA) for emulsion EP0 and EP; isotonicity was achieved with glycerol, 2.5% (w/w) (Pollena-Strem, Dabrowa, Poland) and filled up to 100% with distilled water (Table I).

**Table I:** Composition and characteristics of the dispersed systems (*n* = 6; mean ± SD; *p* < 0.05, between system without BAC and with BAC)

Emulsion	Composition (% w/w)			pH	Size of oily particles [nm]		Zeta potential (mV)
	Oil	Surfactant	BAC		*Z*-average	PDI	
Submicron emulsion							
E0	10	Egg lecithin 1.2	-	6.71 ± 0.01	320 ± 1.2	0.098	− 68.5 ± 1.3
EP0	10	Polysorbate 80 1.2	-	7.27 ± 0.00	310 ± 2.2	0.099	− 44.7 ± 0.9
E1	10	Egg lecithin 1.2	0.005	6.85 ± 0.01	290 ± 2.4	0.089	− 45.3 ± 1.2
E2	10	Egg lecithin 1.2	0.01	6.80 ± 0.01	360 ± 3.3	0.121	− 27.6 ± 0.8
E3	10	Egg lecithin 1.2	0.02	6.87 ± 0.00	370 ± 4.0	0.101	− 8.3 ± 1.0
EP	10	Polysorbate 80 1.2	0.02	7.35 ± 0.01	870 ± 2.7	0.132	+ 6.6 ± 1.1
Traditional emulsion							
tEP	10	Polysorbate 80 1.2	0.02	7.31 ± 0.01	4620 ± 4.4	0.243	+ 8.53 ± 0.9
Aqueous lecithin dispersion							
WLD0	-	Egg lecithin 1.2	-	6.11 ± 0.01	77 ± 1.0	0.092	− 36.8 ± 1.6
WLD	-	Egg lecithin 1.2	0.02	6.49 ± 0.00	82 ± 1.1	0.103	− 27.3 ± 1.9
Aqueous dispersion of nanospheres							
NLC0	45	PlantaCare 2000 5.0	-	6.89 ± 0.01	330 ± 2.3	0.142	− 59.3 ± 1.1
NLC1	45	PlantaCare 2000 5.0	0.005	6.85 ± 0.01	350 ± 1.9	0.138	− 62.5 ± 0.9
NLC2	45	PlantaCare 2000 5.0	0.01	6.82 ± 0.00	340 ± 1.7	0.135	− 60.8 ± 1.3
NLC3	45	PlantaCare 2000 5.0	0.02	6.80 ± 0.01	360 ± 2.1	0.122	− 58.1 ± 0.9

Traditional emulsions (tEP) consisted of soya-bean oil (10% w/w), polysorbate 80 (1.2% w/w), glycerol (2.5% w/w) and filled up to 100% with distilled water were prepared in the similar method as submicron emulsion but without the high-pressure homogenization process (Table I).

Aqueous lecithin dispersion (WLD) was prepared according to methods prepared in our department by dispersing the egg lecithin in the water and filled up to 100% with distilled water and then homogenize using a high-sheer mixer (Table I). The initial dispersion was filtered (0.45 μm Durapore filter, Millipore, Bedford, MA, USA).

Dispersion of lipospheres (lipid nanospheres, NLC), consisted of current black oil (25.0% w/w), beeswax (18.0% w/w), C8–C16 fatty alcohol polyglycoside (PlantaCare 2000) (5.0% w/w), coenzyme Q10 (2.5% w/w), and distilled water up to 100%, was obtained from FU, Berlin, Germany (Table I).

All dispersions were thermally sterilized by autoclaving (121°C, 15 min) and were stored in glass vials with teflon-lined stoppers, at 4°C.

### Adding of benzalkonium chloride to the investigated systems

The antimicrobial agent used in the study was benzalkonium chloride (FeF Chemicals A/S, Køge, Denmark). It was added in a concentration of 0.05 and 0.1 mg/g to the submicron emulsions (E1 and E2, respectively) and 0.2 mg/g to the submicron emulsion E3 and EP as well as to the traditional emulsion tEP, *de novo* by dissolving in the pre-emulsion before high-pressure homogenization. Benzalkonium chloride was added *ex tempore* to nanospheres in a concentration of 0.05 mg/g (NLC 1) by stirring with a glass stirrer (150 rpm, 20 min) (Table I). The second method of adding benzalkonium chloride (0.1 mg/g and 0.2 mg/g) to NLC was *de novo* during the melting of a solid lipid (80°C) (NLC 2 and NLC 3, respectively). Benzalkonium chloride (0.2 mg/g) was added to aqueous lecithin dispersion (WLD) before stirring with a magnetic stirrer.

### Physicochemical characterization of the formulations

The particle size and polydispersity index (PDI) of all tested formulations were measured by photon correlation spectroscopy (PCS), using a Zetasizer Nano ZS90 (Malvern, Malvern, UK). Results from the PCS were expressed in terms of the *Z*-average (diameter). The pH was analyzed with a pH meter (350 type, Orion, Beverly, USA) by immersion of an Ag/AgCl electrode in the emulsion. Zeta potential was determined from the electrophoretic mobility using a Zetasizer Nano ZS (Malvern Instr., Malvern, UK) at 25°C. Prior to the size and zeta measurements, all samples were diluted (1:100) in filtered, demineralized water. The average value (±SD) of at least six measurements was reported.

### Obtaining the aqueous phase (*wlm*) and liposomes/micelles-free aqueous phase (*w*) of dispersed systems

The aqueous phases of the dispersed systems containing liposomes and micelles (*wlm*) were separated by an ultracentrifugation process (ultracentrifuge UP 65, VEB MLV, Engelsdorf, Germany) at 38,000 rpm (14,7000×*g*) for 4 h (25°C). The collected aqueous phase (*wlm*) was opalescent due to liposomal/micellar dispersion, and subsequently, it was subjected to centrifugal ultrafiltration using a Microcon YM-100 filter (Millipore, Bedford, USA) with filters of NMWL 100 kDa, what resulted in a liposomes/micelles-free, transparent aqueous phase (*w*). For NLC only, the ultrafiltration method was performed. The ultrafiltration procedure was validated for the recovery of benzalkonium chloride.

### Quantitative analysis of the benzalkonium chloride in the aqueous phases

**Fig 1:**
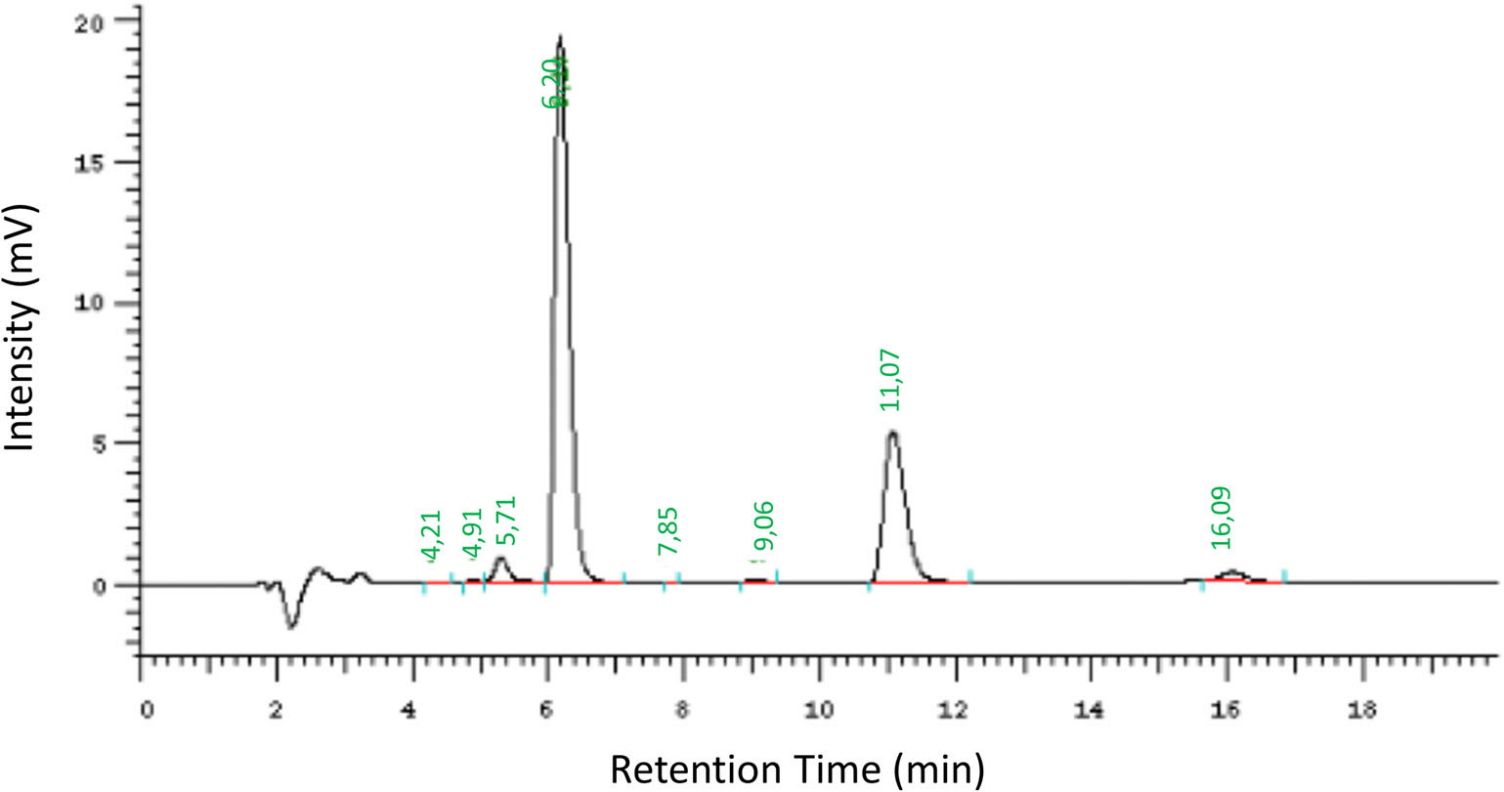
Quantitative determination of BAC content in emulsion E1 by HPLC

A quantitative analysis of the total content of benzalkonium chloride in the separated aqueous phases (*wlm*) as well as in the liposomes/micelles-free aqueous phase (*w*) was performed using an HPLC apparatus (Merck-Hitachi, Darmstadt, Germany) equipped with a C18 column (Lichrocart 5 μm pH = 1, Merck) and a UV-Vis detector at 220 nm. A mixture of acetonitrile and 0.05 M ammonium dihydrogen orthophosphate pH = 2.5 (70:30 v/v) was used as a mobile phase. Prior to the injection, the samples of the aqueous phases (*wlm*) were diluted with acetonitrile due to opalescence and the samples of the liposomes/micelles-free aqueous phase (*w*) were diluted with distilled water. The isolated aqueous phases were injected onto the chromatographic column in order to determine BAC concentrations (Fig. 1).

The method was validated and precision, linearity, specificity, detection, and quantity limits were established.

The specificity of the method for the assay of BAC in the presence of other components of dispersions was evaluated by the comparison of the chromatograms obtained from each dispersion (emulsions, WLD, nanoparticles) containing the standard solution of BAC with the same dispersions without BAC (placebo).

The linearity was evaluated on three different days, against BAC standards with five concentration levels, in the range of 0.5–50 μg/mL. The linearity of the method was determined by a linear regression analysis of the values obtained experimentally with Excel (Microsoft) software.

Precision was considered at two levels: repeatability and intermediate precision. It was determined by intra- and inter-day assays. Stock solutions of BAC were prepared and aliquots were taken to prepare solutions at three levels of concentration: 80%, 100%, and 120% of the sample work concentration. The precision of the method was assessed by the SD and RSD of the values obtained experimentally over three consecutive days. Validation parameters of HPLC method were precision RDS 1.84%, linearity range 0.5–50, and *R* = 0.9998. Each sample was analyzed three times.

### Recovery of benzalkonium chloride after ultrafiltration and ultracentrifugation

The aim of the test was to study if BAC adsorbs on the surface of a Microcon filter during the ultrafiltration process as well as on the surface of tubes during high-speed ultracentrifugation. The aqueous solutions at a known concentration of benzalkonium chloride (similar concentration to determine in aqueous phases, 10 μg/ml and above critical micellar concentration of BAC, 10 mg/ml) were ultrafiltrated three times through the Microcon YM-100 filter (Millipore, Bedford, USA), the same filter which was used for the obtained liposomes/micelles-free aqueous phase (*w*). After each ultrafiltration, the ultrafiltrate was collected in Eppendorf tubes and the content of benzalkonium chloride was analyzed immediately and after 12 h of storage at room temperature by the HPLC method. The aqueous solutions at the same concentration of benzalkonium chloride as above were ultracentrifugated at 38,000 rpm (14,7000×*g*) for 4 h (25°C) in polycarbonate tubes (the same as the dispersions) and the content of benzalkonium chloride was analyzed immediately and after 12 h of storage at room temperature by the HPLC method.

All results were compared with the initial solution of BAC and percentage of recovery of BAC after ultrafiltration as well as after ultracentrifugation was calculated.

### Distribution of benzalkonium chloride between phases of submicron emulsions

Using the concentrations of benzalkonium chloride determined in the *w* and wml phases as well as partition coefficient of BAC between oil and water (*K*_o/w_ was experimentally determined and was 1.1) the amounts of benzalkonium chloride presented in the oily phase and in the lecithin-rich interphase (mesophase) were calculated. Equations 1–5 presented in our previous work (4) and proposed by Han and Washington (2) were employed.

1 $$ {F}_w=\frac{C_w{V}_w}{m} $$

2 $$ {F}_{ml}=\frac{\left({C}_{uc}-{C}_w\right){V}_w}{m} $$

3 $$ {F}_{o+i}=1-\left({F}_w+{F}_i\right) $$

4 $$ {F}_o=\frac{C_w{V}_oP}{m} $$

5 $$ {F}_i={F}_{o+1}-{F}_o $$

where

*F*_w_, *F*_ml_, *F*_*o*_ and *F*_i_: fractions of BAC in the aqueous phase (obtained by ultrafiltration), micellar/liposomal and oily phases and in interphase, respectively
C_w_, C_uc_: concentration of BAC in the aqueous phase obtained by ultrafiltration and ultracentrifugation, respectively
V_w_, V_o_: volume fraction of the aqueous phase and oily phase, respectively
*m*: total mass of BAC in emulsion
*P*: partition coefficient of BAC between oil and water (K_o/w_ = 1.1)

### Statistical analysis

All experimental results obtained are presented as mean (*n* = 6) and standard deviation (SD). The results were evaluated using the nonparametric ANOVA Friedman test. Statistica 13 software (StatSoft, Kraków, Poland) was used in all data analysis. Values of *p* < 0.05 were considered statistically significant.

## RESULTS

### Characterization of dispersed system

**Fig. 2:**
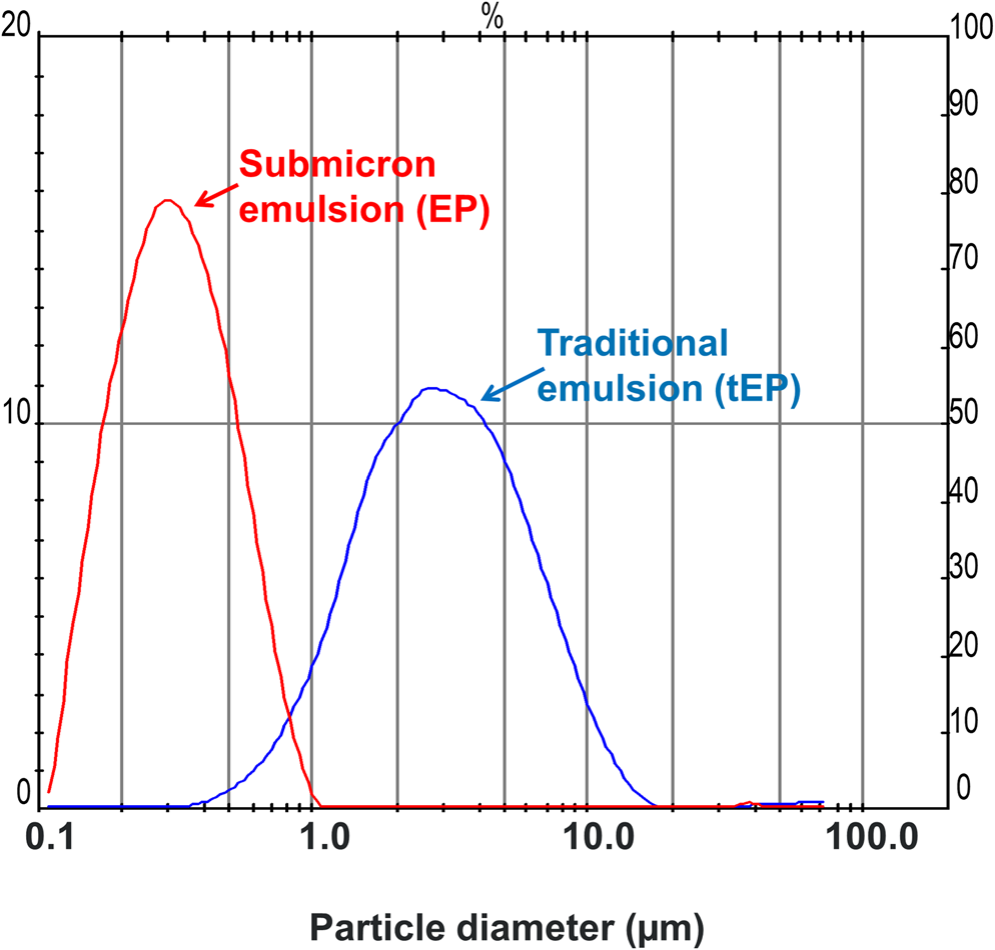
Distribution of oil droplets of emulsions submicron *vs* traditional

Table I presents the characteristics of the prepared formulations. Egg lecithin as well as polysorbate 80 allowed obtaining the submicron size (*Z*-average 320 nm, PDI 0.098) of the preservative-free lipid emulsions (E0 and EP0). The size of emulsion stabilized with lecithin is dose-dependent (V shape): the size first decreased and then increased with the increase of the BAC concentration. There was statistically significant (*p* < 0.05) differences in the size of oily droplets in emulsions stabilized with egg lecithin containing benzalkonium chloride (E1–E3) in comparison with the emulsion without this preservative (Table I). The smallest concentration of BAC (0.005%) caused little decrease in the size of oil droplets (about 30 nm). A higher concentration of BAC resulted in increasing the size of oily droplets (the higher concentration, the higher oil droplets increased), but all emulsions were still in submicron size. The emulsion with polysorbate 80 and BAC (EP) also differed significantly (*p* < 0.05) from the preservative-free emulsion (EP) with respect of the oily droplet size distribution—a statistically significant increase (*p* < 0.05) of oily droplets was noticed (*Z*-average 870 nm, PDI 0.132). The traditional emulsion stabilized with polysorbate 80 (tEP) characterized the non-submicron size of oily droplets (*Z*-average was 4.62 μm, PDI 0.243) because the high-pressure homogenization process hadn’t been used during its preparation (Table I, Fig. 2) but it was stable and phase separation was not observed.

Aqueous lecithin dispersions had a statistically significant (*p* < 0.05) smaller size of dispersed particles (*Z*-average was 77 nm, PDI was 0.092) in comparison with the submicron emulsion due to the lack of the oily phase. The presence of benzalkonium chloride did not significantly change the size of dispersed particles (WLD0 *vs* WLD, Table I).

The size of lipid particles of nanospheres (NLC) was similar to submicron emulsions (*Z*-average 330 nm, PDI 0.142) and there was no statistically significant (*p* < 0.05) influence of the presence and the concentration of benzalkonium chloride (Table I).

Results of pH measurements summarized in Table I showed that the presence of benzalkonium chloride has no influence on the pH value of investigated dispersed systems. The pH of aqueous lecithin dispersions was characterized at about 6.4, whereas nanospheres at about 6.8. For emulsions stabilized with lecithin, the pH was about 6.8 after the thermal sterilization process, whereas the initial pH was about 8.0. A decrease in the pH value after autoclaving for emulsion (the pH drop amounted to about 1 unit from the initial value). As indicated in the literature (21), thermal sterilization of phospholipid dispersions can induce the hydrolysis of phosphatidylcholine and an increase in free fatty acid content, lowering the pH of the preparation. The minor pH reduction (to a value of 7.3) determined for the emulsions stabilized with polysorbate instead of lecithin (EP and tEP), resulted in using the other type of surfactant without phospholipids.

**Fig. 3:**
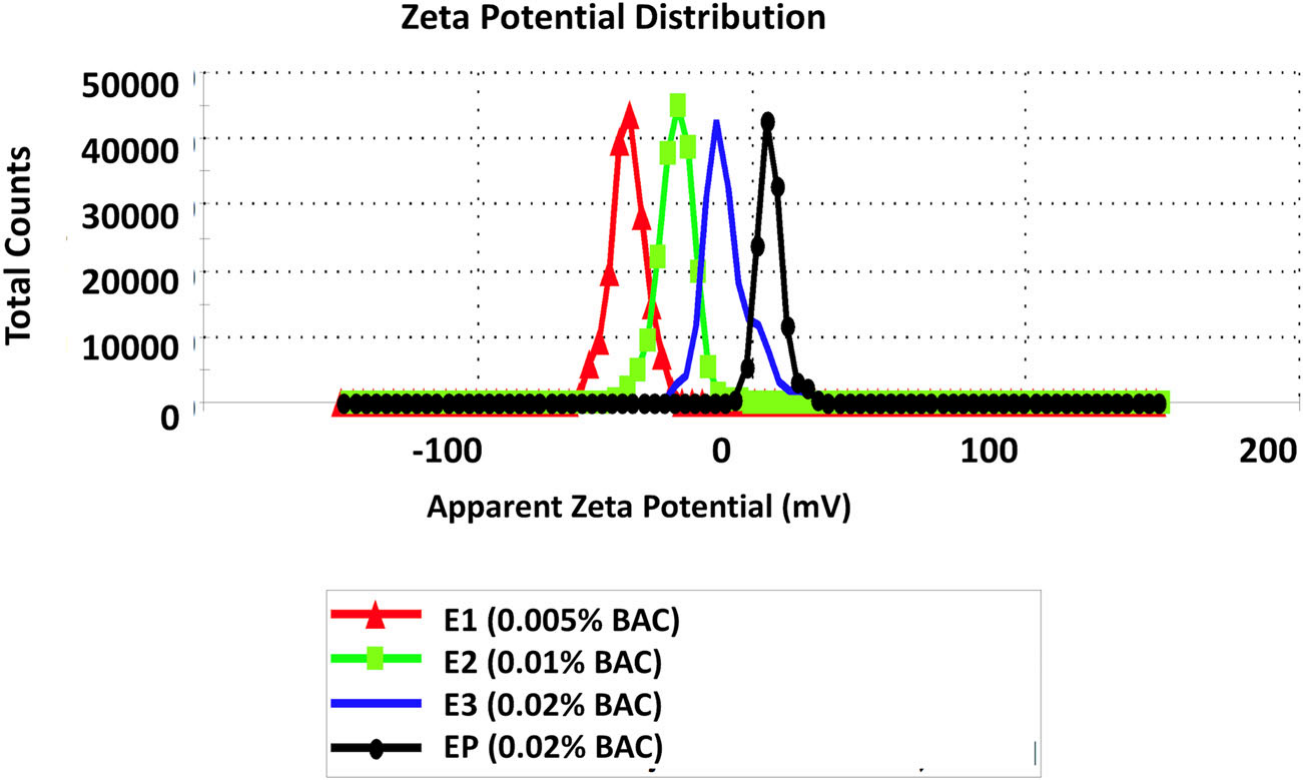
Zeta potential (mV) of submicron emulsion with a different concentration of BAC

Zeta potential measured in the submicron emulsion with lecithin was − 69 mV and in the emulsion with polysorbate was − 45 mV (Table I). Such negative charge should prevent particles from aggregation (22). In the presence of increasing of benzalkonium chloride in submicron emulsions, significant (*p* < 0.05) decrease of negative zeta potential was observed from − 68 to −8.3 mV (Table I). In emulsion stabilized with polysorbate 80, increasing the content of benzalkonium chloride caused inversion of the negative charge of zeta potential for positive (+ 6.6 mV, Fig. 3). A similar situation was observed in the traditional emulsion stabilized with polysorbate where zeta potential was + 8.5 mV. This very interesting observation can be explained by studying the surface parameters of the 2-component mixed surfactant (lecithin or polysorbate 80) and preservative (additive) films at the aqueous solution (23). The phase behavior of cationic/anionic surfactant mixtures strongly depends on the molar ratio and actual concentration of the surfactants. Cationic surfactants have a greater tendency to be incorporated in mixed micelles than anionic ones. The addition of small amounts of cationic surfactant to the anionic surfactant, near or above its critical micellar concentration, and vice versa, results in a shift of the critical micellar concentration of the surfactant in excess toward the lower concentration. Two main factors are responsible for the lowering of the critical micellar concentration: an increase in the entropy of mixing of the surfactant with opposite charge and a decrease in the electrical work of micellization due to the decrease of the surface charge density caused by the solubilized surfactant of opposite charge. This explained why in the presence of BAC in emulsion stabilized with polysorbate 80, positive potential zeta was noticed.

**Fig. 4:**
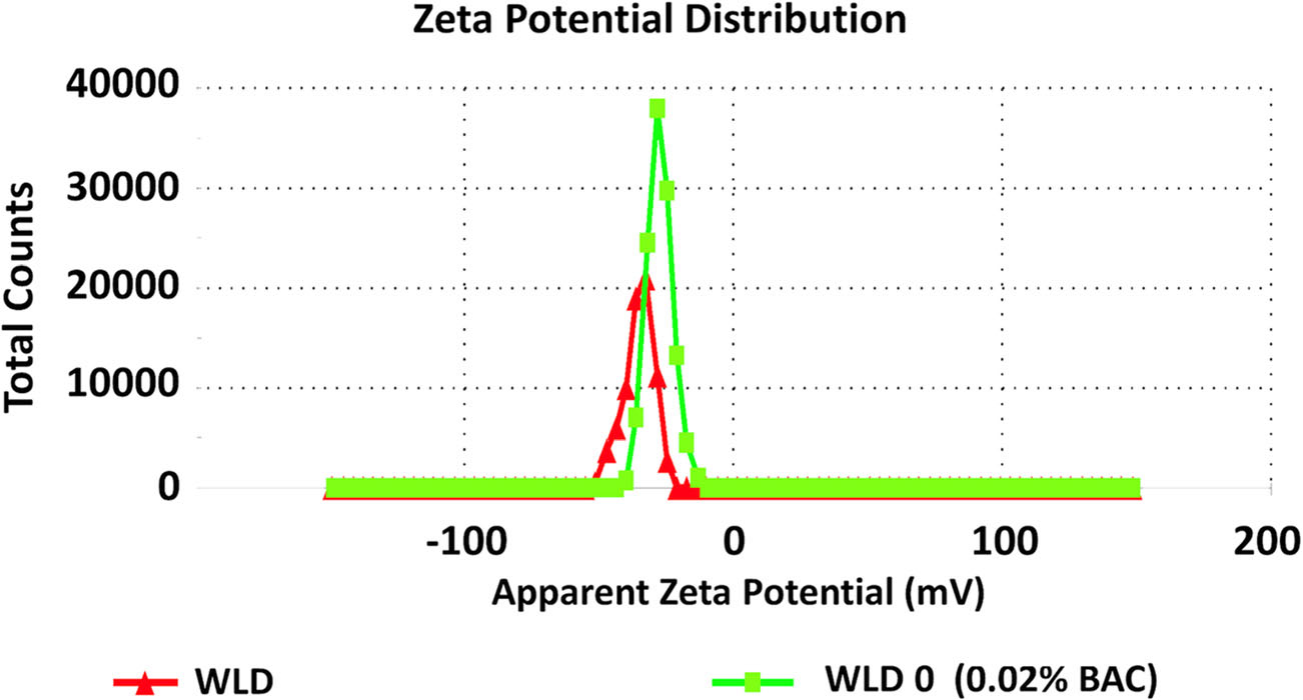
Zeta potential (mV) of aqueous lecithin dispersion—the influence of the presence of BAC

**Fig. 5:**
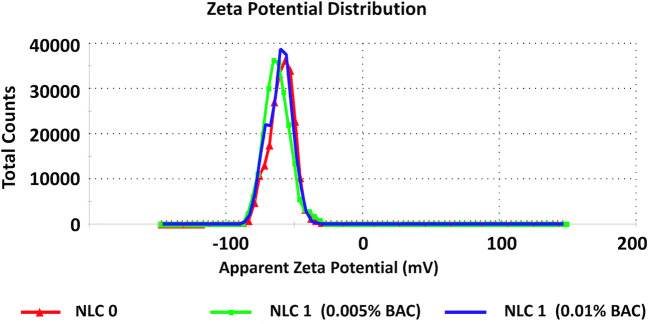
Zeta potential (mV) of dispersion of nanospheres—the influence of the presence of BAC

In aqueous lecithin dispersion, the presence of BAC caused a statistically significant decrease in the negative zeta potential from − 37 to − 27 mV (Fig. 4). However, in the dispersion of nanospheres, no changes in the presence of BAC were noticed (Table I, Fig. 5).

### Fraction of BAC in aqueous and aqueous–liposomal–micellar phases

**Fig. 6:**
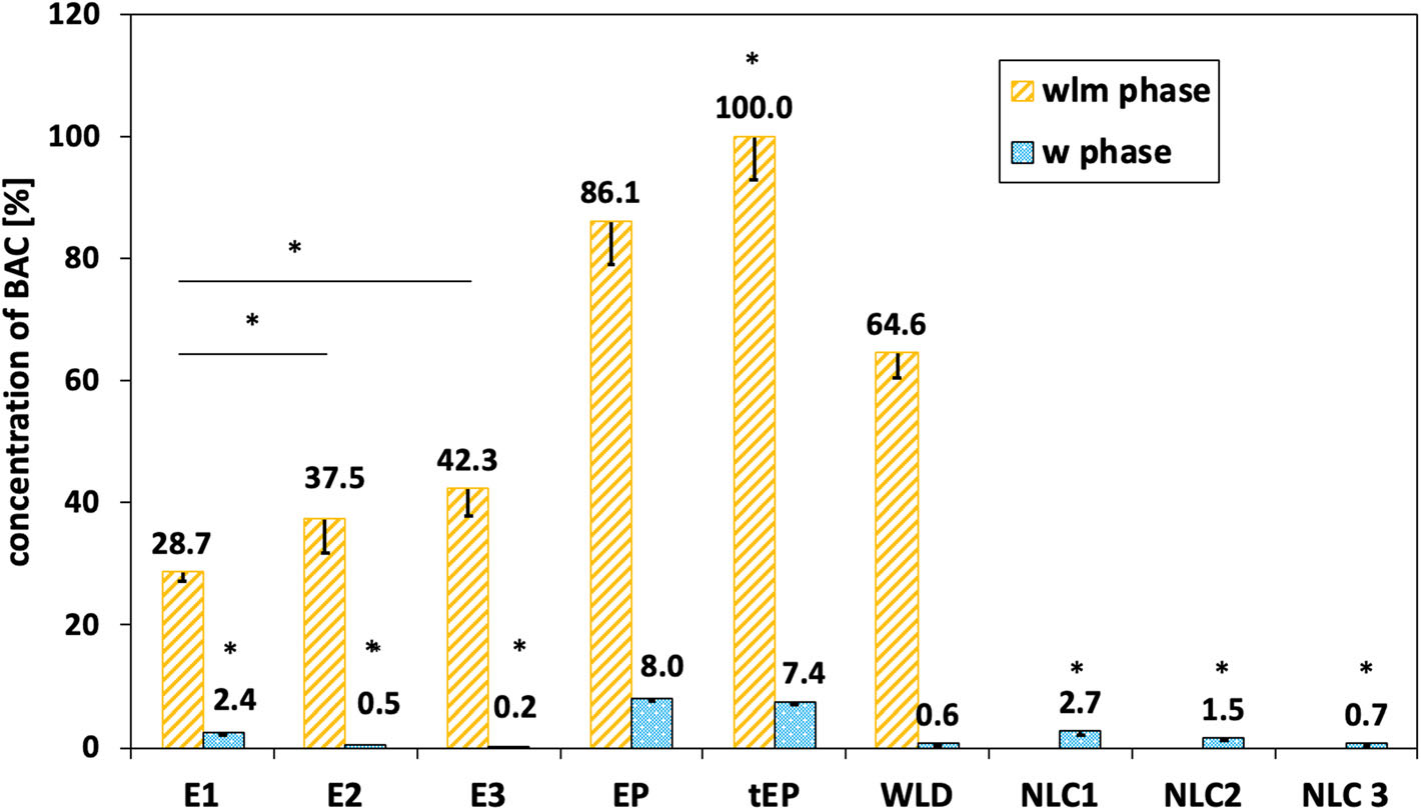
Concentration (% of total content) of benzalkonium chloride in aqueous–liposomal–micellar (*wlm*) and aqueous (*w*) phases of dispersed systems. The mean and standard error of mean in each type of dispersion, *n* = 6. The asterisk indicates significant differences, *p < 0.05 between each type of dispersion

The fractions of benzalkonium chloride determined in the aqueous phases of formulations obtained by ultracentrifugation (aqueous–liposomal–micellar, *wlm* phase) and by ultracentrifugation and then ultrafiltration (aqueous, “*w*” phase) methods were presented in Fig. 6. Fraction *w* given by ultracentrifugation and then the ultrafiltration method represented the free preservative active against microorganisms. Depending on the type of formulations and concentration of BAC, the fraction of this preservative was various. The content of benzalkonium chloride in the aqueous–liposomal–micellar phase (*wlm*) obtained by ultracentrifugation was increased with increasing the initial concentration of this preservative in submicron emulsions with egg lecithin (E1, E2, E3) from 29 to 42%. Significant statistical differences (*p* < 0.05) were noticed between the concentration of BAC in the wlm phase of emulsion E1 and E2 as well as E1 and E3. However, despite increasing the total concentration of benzalkonium chloride in emulsions, a fraction of BAC in the aqueous phase (active form) was very low (approximately 1%) and was decreasing from 2.4 up to 0.2%. Significant statistical differences (*p* < 0.05) were noticed between the concentration of BAC in the aqueous phase of emulsion E1 and E2 and E3. Probably the constant concentration of benzalkonium chloride in the aqueous phase was related with a critical micellar concentration of these binary systems (lecithin and benzalkonium chloride), which was lower than in benzalkonium chloride solution (0.17%) (23).

In the emulsion stabilized with polysorbate 80, the concentration of benzalkonium chloride in the wlm phase was significantly (*p* < 0.05) higher than in emulsion with lecithin: 86% in the submicron emulsion and 100% in the traditional emulsion (tEP) in comparison with emulsion stabilized with egg lecithin (E3, Fig. 6). Significant statistical differences (*p* < 0.05) were noticed between the concentration of BAC in the wlm phase of submicron and traditional emulsion. The traditional emulsion differs from submicron emulsion the internal structures and interphase. The total localization of BAC in the wlm phase of traditional emulsion means that internal structures such as micelles and liposomes are bigger than in the submicron emulsion and entrap BAC inside. An increase in the presence of polysorbate 80 was also observed in the aqueous phase. In the presence of polysorbate 80 in emulsions was discovered about 8% of benzalkonium chloride despite differences in oily droplet size–submicron emulsion (EP) *versus* the traditional emulsion (tEP) (Fig. 6). However, no significant statistical differences (*p* < 0.05) were noticed between the concentration of BAC in the aqueous phase of submicron and traditional emulsion. It is probably caused by grated entrapped of benzalkonium chloride in no ultrafiltered submicron structures created with lecithin than polysorbate.

Using dispersion of lecithin, without oily phase, (WLD) allowed increasing the amount of benzalkonium chloride in the wlm phase to 65% and thrice increased in the aqueous phase (0.6%) in comparison with the submicron emulsion containing the same amount of lecithin (E3; Fig. 6).

Due to much more turbidity of the *wlm* phase of nanospheres, only the concentration of benzalkonium chloride in the aqueous phase (*w*) obtained by ultracentrifugation and ultrafiltration method was performed. A very small concentration of BAC (below 3%) was analyzed in the *w* phase (Fig. 6). The dose-depending effect similar to the emulsions stabilized with lecithin was observed. Increasing the total concentration of benzalkonium chloride from 0.05 to 0.2 mg/g in nanospheres caused statistical differences (*p* < 0.05) decreasing its amount in the *w* phase from 2.7 to 0.7% (Fig. 6).

All results in dispersed systems (concentration of benzalkonium chloride in the *wlm* and *w* phases) were referenced to the total concentration of this preservative in dispersed systems, which was experimentally recovered 92–100% for all dispersed systems.

### Recovery of BAC after ultrafiltration and ultracentrifugation

The ultrafiltration as well as the ultracentrifugation of benzalkonium chloride solution under and below the critical micellar concentration of this preservative was carried out. The results showed no adsorption of submicron structures created by benzalkonium chloride on the ultrafiltration filter Microcon YM-100 as well as on the polycarbonate tubes used in ultracentrifugation. After ultrafiltration, and also the ultracentrifugation of solutions of benzalkonium chloride at concentrations of 10 μg/ml and 10 mg/ml, 97% and 98% benzalkonium chloride were recovered, respectively. This experiment indicated that such a small content of benzalkonium chloride in the aqueous phase of submicron dispersed systems was probably caused by the interaction of this preservative and surfactants presented in these dispersions and created some grated, internal structures which were captured on the ultrafiltration filter.

## DISCUSSION

Modern submicron dispersed systems such as submicron emulsions, which are already used as drug carriers, as well as potential drug carriers aqueous lecithin dispersions (WLD) and suspensions of lipospheres (nanostructured lipid carriers, NLC), were the subject of the presented studies. The characteristic of investigated submicron systems is the presence of phospholipids of lecithin, which in submicron emulsions cover liquid lipid–soya bean oil and in aqueous lecithin dispersions build microparticles with unknown internal structure was evaluated. Aqueous lecithin dispersions differ from submicron emulsions in the lack of oily phase. Nanostructured lipid carriers (lipospheres) consist of solid lipid and different emulsifier—C8–C16 fatty alcohol polyglycoside (PlantaCare 2000). Emulsions stabilized with polysorbate 80 instead of lecithin were studied to compare the influence of phospholipids on the distribution of BAC between phases of submicron emulsion. To investigate the internal structures of the types of dispersions and to compare the influence of the composition on the nanostructure of the dispersed phase, a cryo-electron transmission microscope was used and the results were presented in my previous work (24). It should be noted that microscopic examinations confirmed the results obtained during instrumental size determinations by the PCS method. The smallest in size and most uniform were vesicles suspended in the WLD formulation. Regardless of the differences in the composition of the emulsions, their microscopic images revealed the presence of particles of 2 types: oil droplets (dark spheres) and vesicles (mainly SUV), which number depended on the lecithin content. Replacement of lecithin with polysorbate 80 resulted in a decrease in the number of small liposomes in the formulation.

Benzalkonium chloride was chosen as a preservative—a quaternary ammonium compound which is used in pharmaceutical formulations as an antimicrobial preservative. Benzalkonium chloride is usually nonirritating, nonsensitizing, and well tolerated in the dilutions normally employed on the skin and mucous membranes. Benzalkonium chloride appears to be the main preservative in ophthalmic preparations on the EU market. It is used as an antimicrobial preservative in numerous medicinal products for the nasal route of administration and in many preparations for inhalation use. Other medicinal products that contain benzalkonium chloride are intended for cutaneous, oral, oromucosal, rectal, vaginal, auricular, intravenous/subcutaneous, and intramuscular/intralesional/intraarticular use. In this study, the concentration of BAC was used from 0.5 up to 2 mg/g, which is recommended in the parenteral formulation (8,25).

The size of oily droplets of emulsion stabilized with lecithin was dose dependent (V shape): the size first decreased and then increased with BAC concentration increase.

The main phases of the submicron emulsions are identified as oil (o), aqueous (w), lecithin rich interphase bound to the oily droplets (called interphase, i) and micelles and liposomes (ml) built in water by an excess of lecithin. In order to elucidate distribution of benzalkonium chloride between these phases, equations were employed using concentrations of this preservative determined in the *w* and wlm phases as well as partition coefficients.

**Fig. 7:**
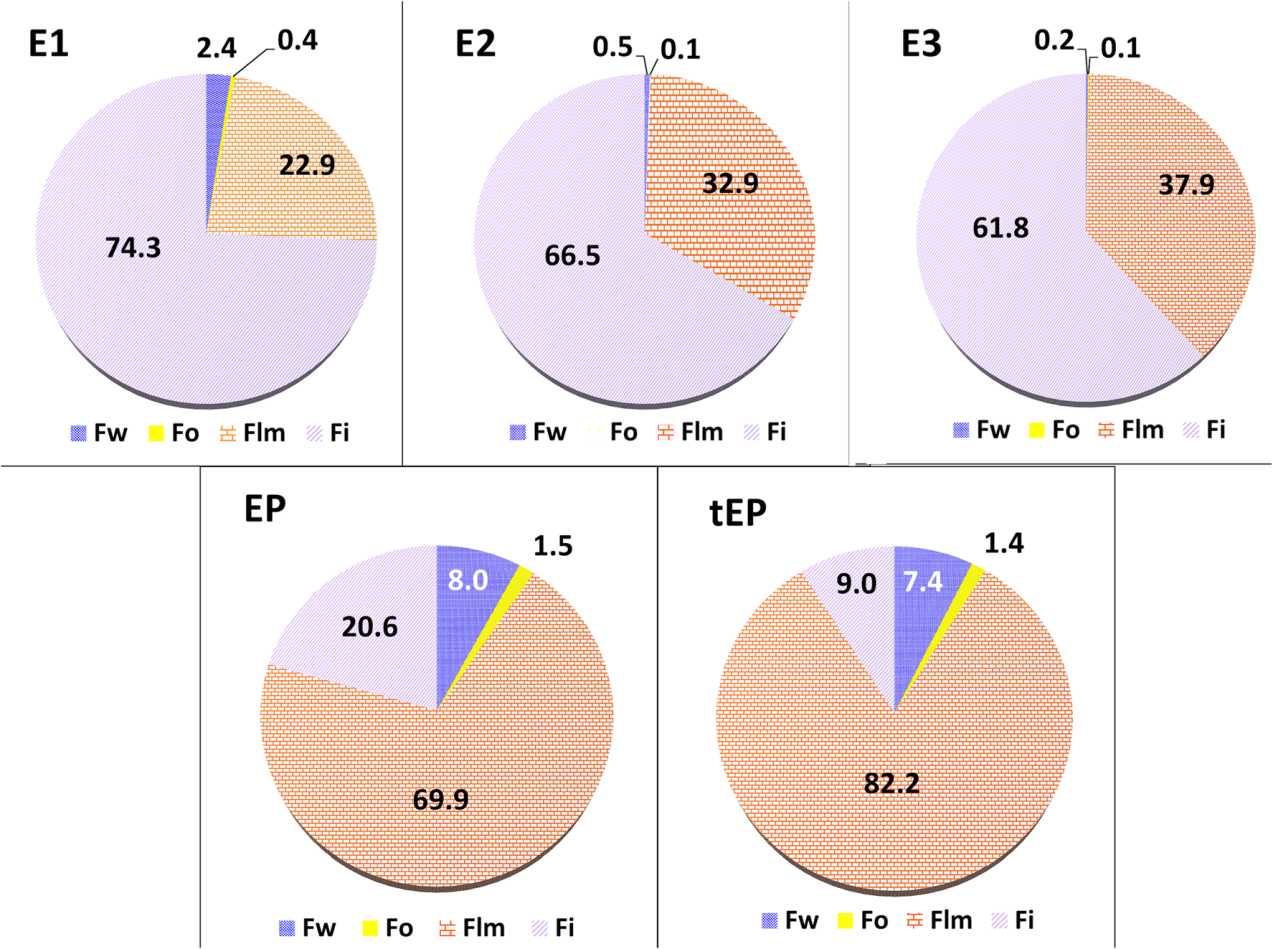
Fraction (% of total content) of benzalkonium chloride (mean ± sd; *n* = 6) between phases of emulsions stabilized with phospholipids or polysorbate (EP, tEP)

Table II and Fig. 7 present the distribution of benzalkonium chloride in all the above phases of the investigated emulsions. Calculation of distribution of BAC into the phase of WLD as well as into NLC was not possible due to lack of oily phase and too much turbidity of wlm phase, respectively. Practically, there was no benzalkonium chloride in the oily phase of submicron emulsions stabilized with egg lecithin (below 0.5%). As it was mentioned, in this kind of emulsion, there was observed a very small fraction (*p* < 0.05) of benzalkonium chloride in the aqueous phase, which is responsible for antimicrobial protection. It was noticed that BAC was mainly localized in the interphase of these emulsions (about 68%). The increase in the total concentration of this preservative in the submicron emulsion from 0.05 to 0.2 mg/g (E1, E2, E3) caused a decreasing fraction of benzalkonium chloride in this phase. When polysorbate was used instead of lecithin, despite the size of oil droplets, a significant increase (*p* < 0.05) of free BAC in the aqueous phase was noticed (up to 8%) and the most content of this preservative was located in the micelles and liposomes phase (about 75%) Fig. 7. It is worthy to know that in liposomal-micellar structures of submicron emulsions stabilized with egg, lecithin was enclosed with about 42% of benzalkonium chloride.

**Table II:** Distribution of benzalkonium chloride between four phases of submicron emulsions (*n* = 6; mean ± SD)

Emulsion	Water (Fw)	Oil (Fo)	Liposomes-micelles (Flm)	Interface (Fi)
E1	2.4 ± 0.15	0.4 ± 0.05	22.9 ± 1.6	74.3 ± 1.7
E2	0.5 ± 0.09	0.1 ± 0.03	32.9 ± 0.9	66.5 ± 1.9
E3	0.2 ± 0.07	0.1 ± 0.04	37.9 ± 1.2	61.8 ± 2.1
EP	8.0 ± 0.9	1.5 ± 0.4	69.9 ± 3.2	20.6 ± 1.8
tEP	7.4 ± 1.3	1.4 ± 0.7	82.2 ± 3.3	9.0 ± 1.7

Such a small distribution of BAC to the aqueous phase of emulsions stabilized with lecithin and WDL and nanospheres is not sufficient for the antimicrobial protection of these systems. The presence of polysorbate 80 in emulsions significantly improved the preservative efficacy test for all standard microorganisms, although not sufficient for *Pseudomonas aeruginosa*. The detailed studies regarding the effectiveness of the antimicrobial preservation test will be presented in another manuscript.

## CONCLUSIONS

The very low concentration of the antimicrobial active form of benzalkonium chloride was analyzed in the aqueous phase of emulsions stabilized with lecithin as well in aqueous lecithin dispersion and nanospheres. Replacement of lecithin with polysorbate 80 in emulsions with polysorbate significantly increased the fraction of benzalkonium chloride in the aqueous phase where microbial growth occurs. BAC can be a potential effective preservative in emulsion stabilized with polysorbate 80, which requires the effectiveness of the antimicrobial preservation test.
